# Safety and Efficacy of Endoscopic Submucosal Dissection in the Management of Gastric Tube Cancers After Esophagectomy: A Systematic Review

**DOI:** 10.7759/cureus.40526

**Published:** 2023-06-16

**Authors:** Rohit Agrawal, James Yang, Saeed Ali, Elie Ghoulam, Hemant Mutneja, Abhishek Bhurwal, Brian Boulay, Edward C Villa

**Affiliations:** 1 Gastroenterology and Hepatology, University of Illinois at Chicago, Chicago, USA; 2 Internal Medicine, University of Illinois at Chicago, Chicago, USA; 3 Internal Medicine, John H. Stroger, Jr. Hospital of Cook County, Chicago, USA; 4 Gastroenterology, Robert Wood Johnson University Hospital, New Brunswick, USA; 5 Gastroenterology and Hepatology, NorthShore University Health System, Evanston, USA

**Keywords:** systematic review and meta-analysis, endoscopic submucosal dissection, gi endoscopy, carcinoma esophagus, gastric tube

## Abstract

Esophagectomy is the proposed standard of care for resectable primary esophageal cancers and recurrent lesions in the reconstructed gastric tube (GT); however, it carries significant morbidity and mortality. Endoscopic submucosal dissection (ESD) has established its role in the management of primary esophageal cancers with growing evidence of its safety in resecting recurrent primary lesions in GT. Our study aims to evaluate the safety and efficacy of ESD in the management of recurrent, localized primary esophageal cancers in GT. We searched PubMed, CENTRAL, EMBASE, Scopus, and clinical trial registries from inception to March 2023 for articles evaluating the safety and efficacy of ESD in the management of recurrent cancerous lesions in GT. Our primary outcome was the en bloc resection rate. Secondary outcomes were curative resection rate, complete resection rate, intra-procedural complication rate, post-procedure complication rate, and five-year survival rate. Seven studies with a total of 165 patients undergoing 192 ESDs were included in the review. The pooled en bloc resection rate was 92.5% (95% CI: 87.7-95.6), which was reported in all seven studies. Pooled complete resection rate was 78.9% (95% CI: 64.5-88.5) per three studies, pooled curative resection rate was 73.9% (95% CI: 63.5-82.2) per four studies, and pooled intra-procedural complication rate was 10.2% (95% CI: 1.5-46.3), which was reported in four studies. Only three studies reported a five-year survival rate that was 65.5% (95% CI: 56.0-73.9). ESD is safe and efficacious in the management of GT cancer after esophagectomy.

## Introduction and background

Esophageal cancer is a major health burden consuming up to half a million lives annually [1]. This seventh most common cancer worldwide is incurable in more than half of the cases, and chemoradiation remains the main stray of therapy for such cancers [2]. However, in around 20% of the cases, esophageal cancers are limited to the submucosa, and such superficial cancers can be surgically resected with or without the need for neo-adjuvant or adjuvant chemoradiation [3]. The incidence of such cancers is increasing globally, and in the United States, its increase is partly attributable to increased endoscopic surveillance [4]. The standard of treatment for such tumors is esophagectomy, which carries high cure rates but is also associated with a significant overall incidence of post-operative complications and mortality rates of up to 4% in high-volume centers [5,6]. Endoscopic therapy is becoming popular for such early cancers and is in fact the most common treatment for T1a lesions in the United States [7,8]. Endoscopic submucosal dissection (ESD) is the preferred endoscopic therapy given the growing evidence supporting higher en bloc resection rates, lower complications, and lower recurrence-free survival rates when compared to piecemeal endoscopic mucosal resection (EMR) [9]. When compared to surgery, long-term outcomes of complete response rates are similar to lower procedure-related complications [10,11].

With increasingly effective treatment, more patients are surviving esophageal cancers with subsequent recurrence of primary cancers in the reconstructed intrathoracic stomach after esophagectomy, which is referred to as gastric tube (GT) [12-14]. Partial or complete GT resection has been historically proposed for the management of such recurrences; however, several retrospective studies, although limited by sample size, have shown the safety and efficacy of ESD in the management of such lesions [15-22]. A meta-analysis by Barakat et al. demonstrated the safety and efficacy of ESD in treating early neoplastic lesions occurring in the surgically altered stomach but did not specifically address the safety of ESD for esophageal cancers in GT [23].

Our study aims to evaluate the safety and efficacy of ESD in the management of primary esophageal cancers in reconstructed GT. Of note, this article was previously presented as a meeting abstract poster at the Digestive Disease Week at San Diego Convention Center, San Diego, California, on May 22, 2022.

## Review

Materials and methods

Standard Cochrane guidelines and PRISMA statement for systematic review and meta-analysis were followed during the review process.

Search Strategy

Three reviewers (R.A., H.M., A.B.) independently searched PubMed, CENTRAL, EMBASE, Scopus, and clinical trial registries using multiple search terms: “endoscopic submucosal dissection,” “early esophageal cancer,” “recurrent esophageal cancer,” “gastric tube cancer,” “gastric tube” and “esophagectomy.” All titles and abstracts were identified by the authors and screened to accrue potentially eligible studies. Then, the same reviewers independently assessed all selected full-text manuscripts for eligibility. Disagreements between the two reviewers were resolved through consensus and after input from the third reviewer and principal investigator.

Types of Studies and Participants

We included studies that were randomized or either retrospective or prospective observational studies that met the following criteria: (1) patients with primary esophageal cancer who were treated with esophagectomy with GT reconstruction, (2) those who had ESD for early GT cancers (GTCs), and (3) where adequate data were presented on outcomes being considered (mentioned below).

Types of Outcome Measures

The primary outcome was the en bloc resection rate (single-piece resection of the lesion). Secondary outcomes included curative resection rate (complete resection with no evidence of lymphovascular invasion), complete resection rate (en bloc resection with neoplasm-free margins), intra-procedural complication rate, post-procedure complication rate, and five-year survival rate [24].

Data Extraction

Two authors (R.A., H.M.) extracted the data from the included studies to collect the participants, baseline characteristics, methods, interventions, and outcomes. The reviewers sorted the data separately in all stages of study collection, data extraction, and quality assessment. All discrepancies found between the two reviewers were resolved with consensus and inputs from other authors.

Study Characteristics and Assessment of Risk of Bias

All nonrandomized studies were evaluated using the Newcastle-Ottawa scale [25]. For each study, we assessed the study design and content. The studies were then graded using a “star system” based on (1) the selection of the study groups, (2) the comparability of the groups, and (3) the ascertainment of the outcome of interest.

Quality assessments were also conducted independently, and discrepancies were resolved by consensus.

Quantitative Data Synthesis

All outcomes were analyzed using the Comprehensive Meta-Analysis software package (Biostat, Englewood, NJ, USA). The final pooled risk estimates were obtained using random effects models. The Cochrane Q and the I^2^ statistics were calculated to assess heterogeneity between studies. P < 0.10 for chi-square test and I^2^ < 20% were interpreted as low-level heterogeneity.

Results

Results of the Search

**Figure 1:**
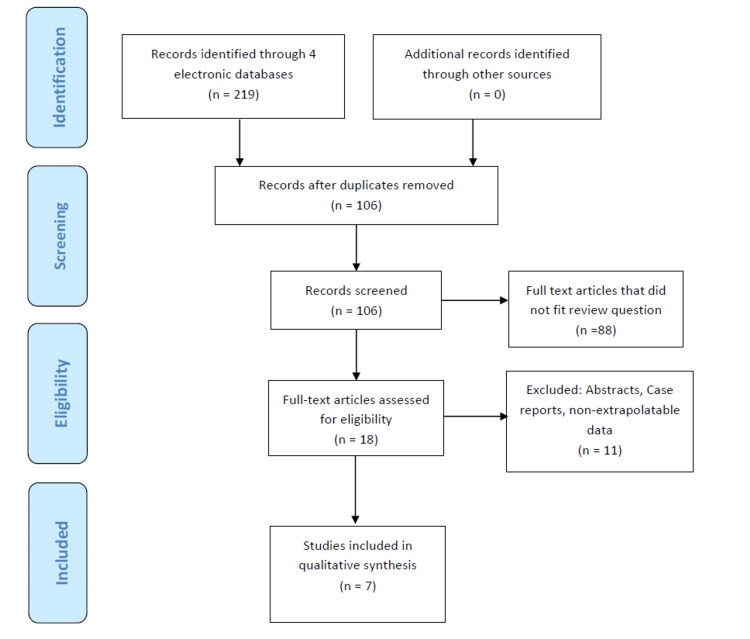
PRISMA flowchart PRISMA, Preferred Reporting Items for Systematic Reviews and Meta-Analyses

The preliminary search yielded 219 titles. We identified 18 potential studies to be included after a review of titles and abstracts. After reading the full text of potentially eligible studies, 11 more were excluded. Seven studies met the inclusion criteria, including a total of 165 patients who underwent 192 ESDs for GTC (Figure 1).

Inter-rater agreement during the review process was high (kappa = 0.7).

*Included Studies * 

Seven studies with a total of 165 patients were included in the review. All the studies were retrospective in design. Out of the seven studies, six were single-center studies and one was a multi-center study. All studies included patients undergoing ESD for GTC. We did not identify any prospective trials. Average age of patients was 70.2 years, and 149 (90.3%) were men. A total of 192 ESDs were performed with an average procedure time of 114.2 minutes. Tumor size ranged from 15 to 26 mm (Table 1). 

**Table 1:** Baseline characteristics of studies included in our analysis GTC, gastric tube cancer

Author	Age (range)	Patients (male %)	No of GTC	Procedure time	Follow-up (months)	Tumor size (mm, range)	En block	Complete	Curative	Survival at 5 years
Satomi et al. [16]	71.5 (57-84)	38 (34)	48	81 (29-334)	28.1 (0.26-134.6)	15 (4-60)	44		38	
Watanabe et al. [17]	72.5 (55-82)	18 (17)	20	87.5 (19-242)	108 (24-264)	16 (8-61)	20	16	16	59.50%
Mukasa et al. [18]	70.5 (65-78)	11 (10)	11	142 (32-445)		26.1 (10-68)	11			70.9%
Nonaka et al. [19]	67.4	51 (47)	79	90 (15- 360)	45.6 (0-145.2)	15 (3-50)	73	59	51	
Tawaraya et al. [20]	70 (61-79)	15 (13)	16	92.9 (24-252)		18.6 (5-42)	16	16	13	68.40%
Bamba et al. [21]	69.5 (58–84)	25 (22)	10	162 (50-375)	45 (12–280)	20.6 (6-52)	9			
Osumi et al. [22]	70.6 (56–80)	7 (6)	8	144 (39-266)	13.5 (8–20)		7			

Bias Assessment

Newcastle-Ottawa scoring system was used for assessment of the quality of the retrospective studies (Table 2).

**Table 2:** Newcastle-Ottawa scoring system for assessment of quality of the retrospective studies

Study	Selection	Comparability	Exposure	Total score of quality
Satomi et al. 2021 [16]	★★★★	★★	★★	8
Watanabe et al. 2019 [17]	★★★	-	★★★	6
Mukasa et al. 2015 [18]	★★★	-	★★	5
Nonaka et al. 2014 [19]	★★★	-	★★★	6
Tawaraya et al. 2014 [20]	★★★	-	★★★	6
Bamba et al. 2010 [21]	★★★★	-	★★	6
Osumi et al. 2009 [22]	★★★	-	★★	5

Outcome Analysis

**Figure 2:**
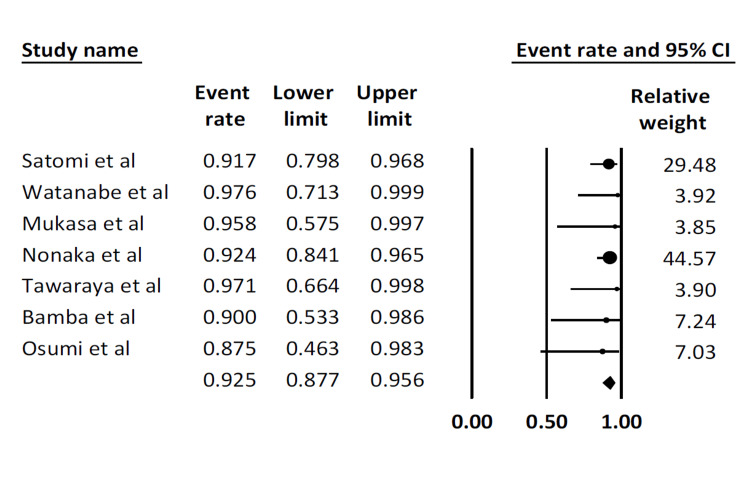
Pooled en bloc resection rate Satomi et al. [16], Watanabe et al. [17], Mukasa et al. [18], Nonaka et al. [19], Tawaraya et al. [20], Bamba et al. [21], Osumi et al. [22]

En bloc resection rate: All seven studies reported this outcome. The pooled en bloc resection rate was 92.5% (95% CI: 87.7-95.6). There was no statistical heterogeneity (I^2^ = 0%) (Figure 2).

**Figure 3:**
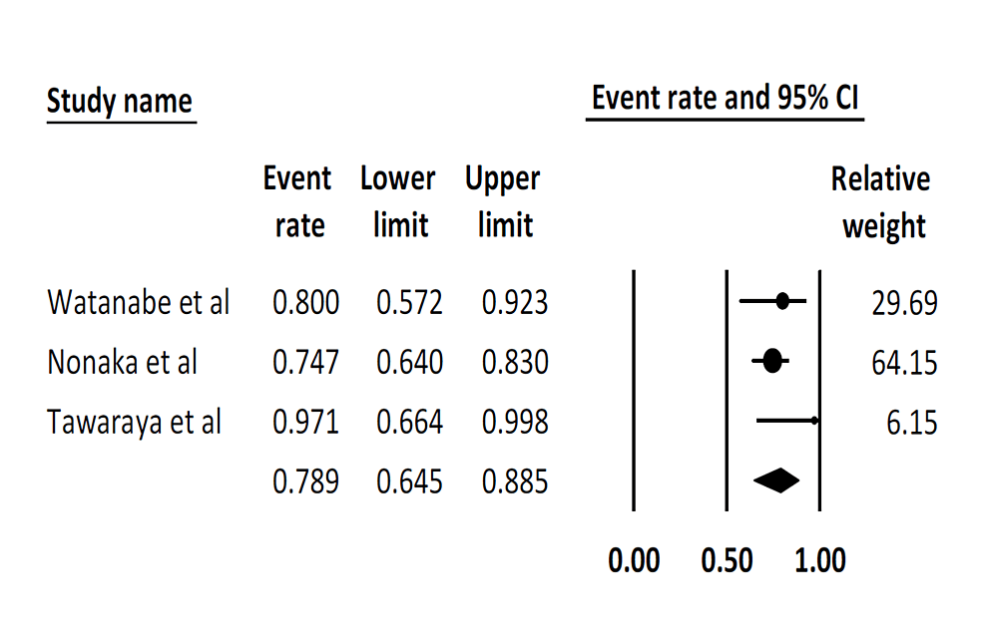
Pooled complete resection rate Watanabe et al. [17], Nonaka et al. [19], Tawaraya et al. [20]

Complete resection rate: This outcome was reported by only three studies. The pooled complete resection rate was 78.9% (95% CI: 64.5-88.5). There was moderate statistical heterogeneity (I^2^ = 30.5%) (Figure 3).

**Figure 4:**
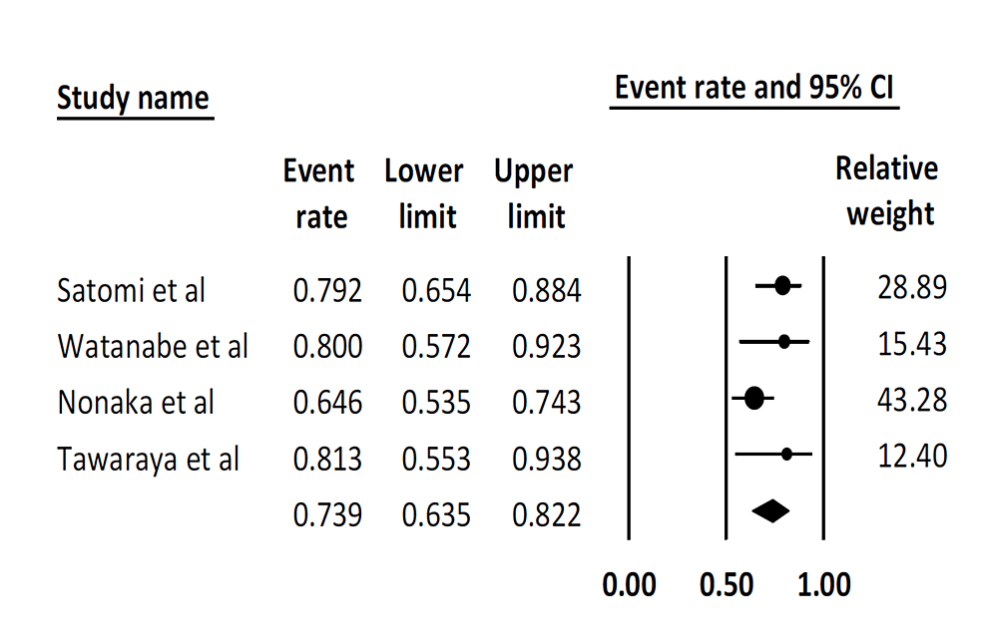
Pooled curative resection rate Satomi et al. [16], Watanabe et al. [17], Nonaka et al. [19], Tawaraya et al. [20]

Curative resection rate: This outcome has been reported in four studies. The pooled curative resection rate was 73.9% (95% CI: 63.5-82.2). There was moderate statistical heterogeneity (I^2^ = 35.3%) (Figure 4).

**Figure 5:**
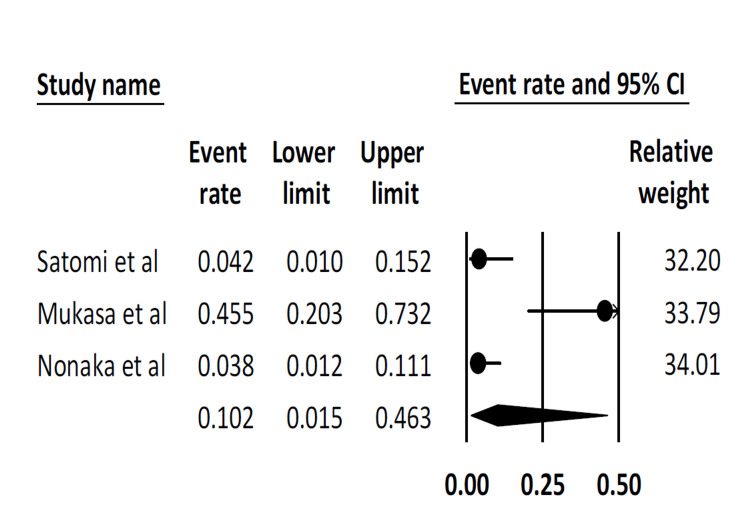
Pooled intra-procedural complication rate Satomi et al. [16], Mukasa et al. [18], Nonaka et al. [19]

Intra-procedural complication rate: This outcome was reported by only three studies. The pooled intra-procedural complication rate was 10.2% (95% CI: 1.5-46.3). There was substantial statistical heterogeneity (I^2^ = 87.3%) (Figure 5).

**Figure 6:**
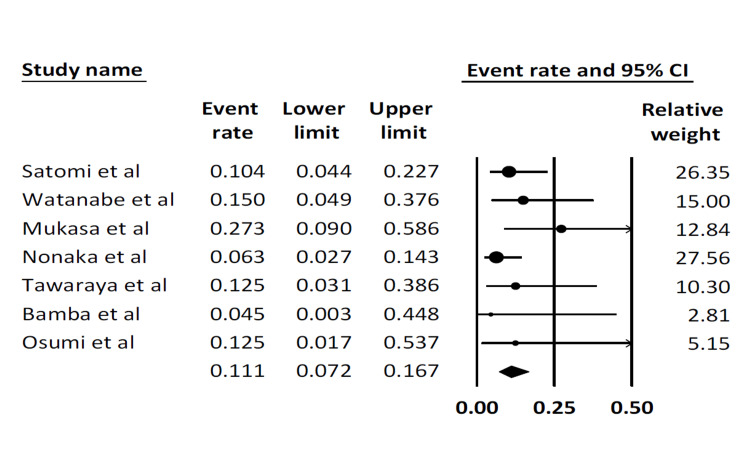
Pooled post-procedure complication rate Satomi et al. [16], Watanabe et al. [17], Mukasa et al. [18], Nonaka et al. [19], Tawaraya et al. [20], Bamba et al. [21], Osumi et al. [22]

Post-procedure complication rate: This outcome was reported by all seven studies. The pooled post-procedure complication rate was 11.1% (95% CI: 7.2-16.7). There was no statistical heterogeneity (I^2^ = 0%) (Figure 6).

**Figure 7:**
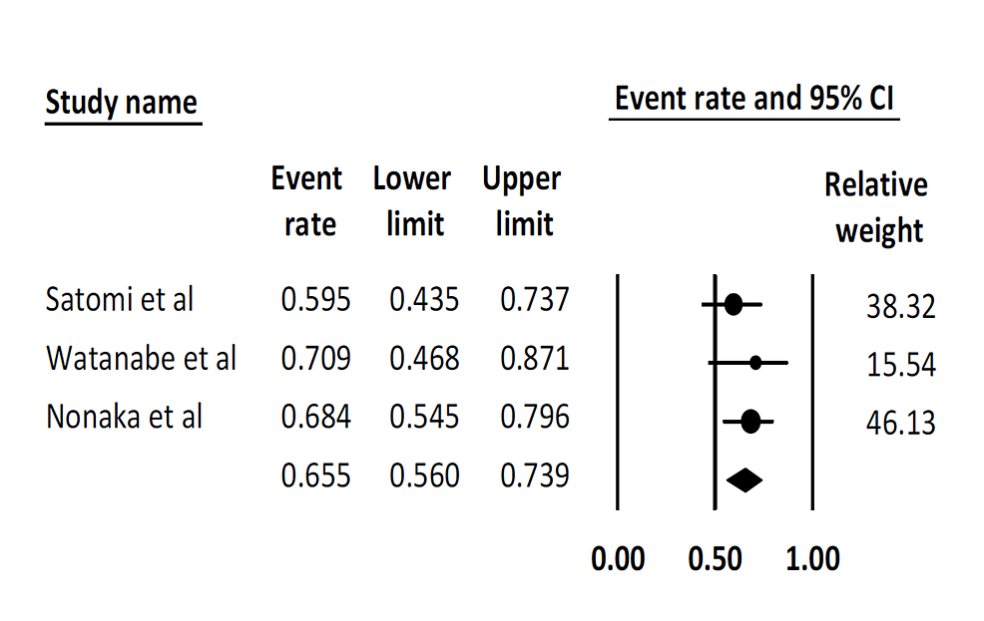
Pooled five-year survival rate Satomi et al. [16], Watanabe et al. [17], Nonaka et al. [19]

Five-year survival rate: This outcome was reported by only three studies. The pooled five-year survival rate was 65.5% (95% CI: 56.0-73.9). There was no statistical heterogeneity (I^2^ = 0%) (Figure 7).

Discussion

As the survival of patients after curative esophagectomy increases, we are likely to encounter more cases of GTC. Data from prospective studies have shown an incidence of approximately 6% for such GTC [26,27]. Although esophagectomy is considered the standard therapy for GTCs, other less invasive and efficacious therapies such as EMR are gaining an increasing role in early neoplasms [27,28]. EMR is being used with other modalities such as radio-frequency ablation, photodynamic therapy, and cryotherapy with curative intent for Barrett’s esophagus and primary esophageal cancers especially when tumors are less than 2 cm in size, limited to the mucosa, and involve less than one-third of circumference [29-31]. When compared to surgery, patients who were treated endoscopically had higher 30-day survival rates [7]. ESD is more commonly being employed for larger esophageal cancers with studies reporting en bloc resection rates of close to 100% with minimal complication [32,33]. Superiority of ESD over EMR has been shown in a retrospective study of patients with squamous cell cancer (SCC) of the esophagus with higher en bloc resection and lower recurrence rates of 1 versus 10% when compared to EMR [34]. ESD for lesions in the reconstructed GT is technically challenging given fluid pooling along with narrow working space and the underlying fibrosis after surgery with interference from staple lines [18,21]. However, based on the studies included in our analysis, it is now being employed for the management of such lesions and has shown favorable outcomes [16-22].

Our analysis demonstrated a pooled en bloc resection rate of 92.5% and a curative resection rate of 73.9%, with low intra-procedural and post-procedural adverse events. We had a five-year survival rate of 65.5%, which is compatible with the existing literature. Nonaka et al. reported a five-year overall survival rate of 68.4% in patients with GTC after esophagectomy undergoing ESD at their center in Japan [19]. We have reported an en bloc resection rate of 92.5%, whereas other studies have en bloc resection rates of 83-100% for primary SCC. Similarly, we have reported pooled complete resection rate of 78.9%, whereas other studies have reported a rate of up to 78-100% for primary SCC of the esophagus [35].

Complication rates of ESD for primary SCC of the esophagus are overall low and have been reported with bleeding up to 1.5%, perforation up to 5%, and strictures up to 6.5% [36]. When compared to surgery, endoscopic therapy has similar median cancer-free survival with significantly lower morbidity [3].

To our knowledge, this is the first systematic review evaluating the safety and efficacy of ESD for the management of GTC after esophagectomy. We conducted a comprehensive literature search and included all relevant studies. We had a decent sample size of 165 patients who had a total of 192 ESDs for the GTC. One of the studies was multi-center and others were single-centered studies. The quality of the included studies was acceptable based on the Newcastle-Ottawa scoring system. And the results were overall compatible with the existing published literature.

Limitations

Our study is a systematic review of retrospective studies, which is inherently limited by bias. Baseline patient characteristics and prior treatment details were not available. Institutional expertise and procedural differences that include but are not limited to types of ESD instruments used could potentially also affect the rates of resection. Also, these procedures were performed in a large, specialized tertiary center, which makes our study prone to selection bias as well and limits the generalizability of results.

## Conclusions

Our study showed that ESD is safe and efficacious in the management of GT cancer after esophagectomy. The pooled en bloc resection rate was 92.5%, pooled complete resection rate was 78.9%, pooled curative resection rate was 73.9%, and pooled intra-procedural complication rate was 10.2%. The five-year survival rate was 65.5%. Our analysis yet again reiterates the growing utility of ESD in the management of cancers including but not limited to surgically altered anatomy.
